# Thrombotic Events in Patients With Paroxysmal Nocturnal Haemoglobinuria Using Eculizumab: A Post‐Marketing Surveillance Sub‐Analysis

**DOI:** 10.1002/jha2.70287

**Published:** 2026-05-26

**Authors:** Takayuki Ikezoe, Tatsuya Kawaguchi, Hideo Hayashi, Akihiko Shimono, Jun‐ichi Nishimura

**Affiliations:** ^1^ Department of Hematology Fukushima Medical University Fukushima Japan; ^2^ Department of Medical Technology Kumamoto Health Science University Kumamoto Japan; ^3^ Medical Affairs Division Alexion Pharma G.K. Tokyo Japan; ^4^ Department of Hematology and Oncology Osaka University Graduate School of Medicine Osaka Japan

**Keywords:** C5 inhibitor, eculizumab, intravascular haemolysis, paroxysmal nocturnal haemoglobinuria, thromboembolic events

## Abstract

**Background:**

Thrombotic events (TEs) account for approximately 40%–67% of deaths in untreated paroxysmal nocturnal haemoglobinuria (PNH). C5 inhibitor treatment (e.g., eculizumab and ravulizumab) greatly reduces the incidence of TEs in patients with PNH, but data on the characteristics of PNH patients experiencing TEs are limited.

**Methods:**

This post‐marketing surveillance sub‐analysis evaluated the incidence of TEs in patients with PNH in Japan.

**Results:**

During eculizumab treatment, 54 TEs occurred in 44/794 patients (5.5%), an incidence of 1.50/100 patient‐years. The most frequent TEs were deep vein thrombosis and cerebral infarction (eight events [14.8%]). A history of TE before eculizumab treatment was associated with TE during eculizumab treatment (*p* = 0.014), and greater cumulative incidences of TEs and multiple TEs during treatment (both *p* = 0.003) versus no history of TE. TEs were more frequent during the antithrombotic agent off‐period (71.8%, 28/39) than during the on‐period (28.2%, 11/39). TEs during eculizumab treatment were observed in patients with high lactate dehydrogenase (LDH) levels, infection events and longer administration intervals. Within the week before TE onset, LDH levels > 1.5 × the upper limit of normal were observed in 8/13 patients with TEs (61.5%), suggesting that intravascular haemolysis is not adequately suppressed in these patients.

**Conclusion:**

Eculizumab helps prevent TEs in PNH. TE events appear to be associated with higher LDH levels, infections and long dosing intervals. Concurrent antithrombotic agent use to prevent TEs in PNH is likely clinically useful, especially in patients with a history of TE.

**Trial Registration:**

The authors have confirmed clinical trial registration is not needed for this submission.

## 1 Introduction

Paroxysmal nocturnal haemoglobinuria (PNH) is a rare, life‐threatening, chronic haematopoietic stem cell disorder with uncontrolled terminal complement activation of blood cells leading to intravascular haemolysis (IVH), thromboembolic events (TEs) and organ damage resulting in premature mortality [1, 2, 3]. PNH presents with haemolytic anaemia, bone marrow failure (BMF), thrombophilia, or a combination of these manifestations. Up to 44% of untreated PNH patients experience TEs [4, 5]. TEs can be fatal, accounting for roughly 40%–67% of PNH‐related deaths before eculizumab introduction, with a 5‐year mortality rate of approximately 30% [4, 6, 7]. The current standard of care for PNH uses C5 inhibitors (e.g., ravulizumab and eculizumab) to inhibit terminal complement activation and consequent IVH, which enables many patients to control their condition by reducing the risk of TEs and other PNH‐related symptoms [8, 9].

Eculizumab is a humanized, monoclonal antibody to C5 that inhibits terminal complement pathway activation, thereby preventing the formation of the C5a, C5b and C5b‐9 membrane attack complex, which is crucial for the IVH process in PNH [5]. Thus, the mechanistic action of this drug in reducing the release of free haemoglobin mitigates TE risk [4, 9]. The efficacy of eculizumab was demonstrated in broad populations of patients with PNH in two Phase 3 trials (TRIUMPH [10] and SHEPHERD [11]), in which intravenous administration of eculizumab decreased lactate dehydrogenase (LDH), a biomarker of IVH and improved haemoglobin levels [12]. Furthermore, long‐term eculizumab administration reduces the risk of TEs in patients with PNH [13]. In the International PNH Registry, Terriou et al. reported a 60% reduction in TE and major adverse vascular event risk during eculizumab treatment versus no eculizumab treatment [14]. Although Asian patients have been reported to have a lower risk of TEs than patients in the United States [15], these patients should also be monitored closely for TE risk. This is especially relevant in cases with LDH levels ≥ 1.5 × upper limit of normal (ULN) because of the possible higher risk of TEs [15] and mortality [4].

Although TEs may be caused by PNH itself, other factors outside of PNH, such as advanced age and lifestyle‐related disease, may contribute to their occurrence [16, 17, 18]. Understanding the characteristics associated with TEs in patients with PNH is crucial, given their potentially fatal outcomes.

Previously, we reported the safety and effectiveness of eculizumab in Japanese patients with PNH based on long‐term follow‐up post‐marketing surveillance (PMS) data [19], where 75/632 (11.9%) patients had a history of TE. Antithrombotic agents were used in 104/632 (16.5%) patients before the commencement of eculizumab and concomitantly used in 94/632 (14.9%) patients during eculizumab treatment [19]. However, the incidence of TEs in Japanese patients with PNH under C5 inhibitor treatment was not evaluated. This sub‐analysis evaluates TEs in patients with PNH during eculizumab treatment and explores the relationship between antithrombotic agent use and underlying conditions.

## 2 Methods

### 2.1 Study Design and Patients

This PMS was a Japanese government‐mandated, observational study to evaluate the safety and effectiveness of eculizumab in the real‐world setting in Japan [19]. The study was conducted from June 1, 2010 (when eculizumab was launched in Japan) to August 31, 2019, during which time case report forms completed by attending physicians were collected annually. All patients diagnosed with PNH using flow cytometry and/or the “Ham's test and sugar‐water test” [20], and treated with eculizumab at least once, were enrolled. The attending physicians determined the treatment based on the approved regimen, which advises a starting dose of 600 mg, with a total of four intravenous infusions (including the initial dose), at 1‐week intervals, followed by an intravenous infusion of 900 mg 1 week later (i.e., 4 weeks after the initial dose), then administered at 2‐week intervals thereafter. Any patients who discontinued eculizumab during the surveillance period were followed for 8 weeks after discontinuation. Patients for whom consent for publication could not be obtained were excluded from the safety analysis set.

### 2.2 Identification of TEs

TEs were identified as any adverse event (AE) reported as thrombosis‐related (e.g., stroke, myocardial infarction, disseminated intravascular coagulation, pulmonary embolism, venous thromboembolism, arterial thrombosis, or Budd–Chiari syndrome) from baseline (the first evaluation before receiving eculizumab treatment) to the end of the PMS. MedDRA/J Version 22.1 was used to classify and tabulate AEs. The incidence (per patient‐year) of TEs and the percentage of patients with TEs during eculizumab treatment were calculated. Patient characteristics were compared between patients with or without TEs and those who experienced multiple TEs under eculizumab treatment.

In patients with a TE, the timing and duration of antithrombotic agent use during eculizumab treatment and its relationship to TE occurrence, as well as the LDH level closest in time prior to TE onset, were evaluated.

### 2.3 Statistical Analysis

In this sub‐analysis, the sample size was predetermined by the number of patients enrolled in the PMS. The safety set included all enrolled patients who received the study drug at least once. Continuous variables were summarised as mean ± standard deviation or median (range). Normally distributed quantitative parameters between groups were assessed using Fisher's exact test or *t*‐test. The cumulative incidence of TEs throughout the observation period, based on whether the patients had a history of a TE or not, was also assessed by Kaplan–Meier analysis.

Missing data were not imputed, and outliers were not considered. The statistical analysis was conducted using SAS version 9.3 (SAS Inc., Cary, NC, USA) or later. Two‐sided *p*‐values (significance level < 0.05) were used.

## 3 Results

### 3.1 Patient Population

Of the 902 patients enrolled in the eculizumab PMS, 794 gave consent for publication and were included in the analysis (Figure S1). At baseline, patients had a median (range) age of 59.0 (9.0–90.0) years, 391/794 (49.2%) were female and 96/794 (12.1%) had a history of TE (Table S1). The median (range) eculizumab treatment duration was 1389.0 (1.0–4215.0) days, with a total exposure of 3609.990 patient‐years.

### 3.2 Incidence of TE‐Related AEs

During eculizumab treatment, 54 TEs occurred in 44/794 patients (5.5%), with an incidence of 1.50/100 patient‐years (Table 1). The most frequently reported TEs were deep vein thrombosis and cerebral infarction (eight events [14.8%] each). Nineteen events (35.2%) were arterial blood TEs, 18 (33.3%) were venous blood TEs and 17 (31.5%) cases were unclassified.

**TABLE 1:** Incidence of TE during eculizumab treatment.

Items	Events, *n*	Incidence (/100 patient‐years)	Patients, *n* (%)
Total	54	1.50	44 (100.0)
Vascular disorders	18	0.50	18(40.9)
Deep vein thrombosis	8	0.22	8 (18.2)
Venous thrombosis in limb	5	0.14	5 (11.4)
Thrombosis	3	0.08	3 (6.8)
Peripheral arterial occlusive disease	2	0.06	2 (4.5)
Nervous system disorders	11	0.30	10 (22.7)
Cerebral infarction	8	0.22	7 (15.9)
Cerebrovascular accident	1	0.03	1 (2.3)
Haemorrhagic cerebral infarction	1	0.03	1 (2.3)
Lacunar infarction	1	0.03	1 (2.3)
Blood and lymphatic system disorders	7	0.19	7 (15.9)
Disseminated intravascular coagulation	6	0.17	6 (13.6)
Heparin‐induced thrombocytopenia	1	0.03	1 (2.3)
Cardiac disorders	6	0.17	5 (11.4)
Acute myocardial infarction	3	0.08	3 (6.8)
Myocardial infarction	1	0.03	1 (2.3)
Cardiac ventricular thrombosis	2	0.06	1 (2.3)
Respiratory, thoracic and mediastinal disorders	3	0.08	3 (6.8)
Pulmonary embolism	3	0.08	3 (6.8)
Hepatobiliary disorders	4	0.11	3 (6.8)
Portal vein thrombosis	2	0.06	2 (4.5)
Budd–Chiari syndrome	1	0.03	1 (2.3)
Hepatic vein thrombosis	1	0.03	1 (2.3)
Injury, poisoning and procedural complications	2	0.06	2 (4.5)
Shunt occlusion	1	0.03	1 (2.3)
Shunt thrombosis	1	0.03	1 (2.3)
Gastrointestinal disorders	1	0.03	1 (2.3)
Haemorrhoids thrombosed	1	0.03	1 (2.3)
Renal and urinary disorders	1	0.03	1 (2.3)
Renal vein thrombosis	1	0.03	1 (2.3)
Pregnancy, puerperium and perinatal conditions	1	0.03	1 (2.3)
Placental infarction	1	0.03	1 (2.3)
MedDRA/J Version (22.1)			
Abbreviation: TE, thrombotic events.			

### 3.3 Characteristics of Patients with or without TEs during Eculizumab Treatment

**FIGURE 1:**
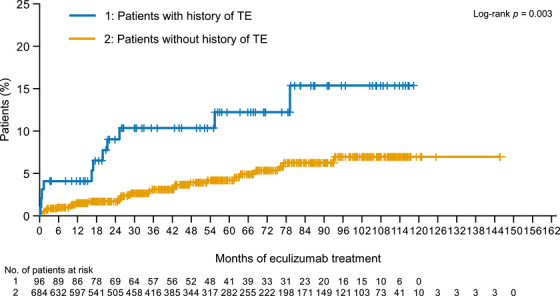
Cumulative incidence of TE (with/without a history of TE). Abbreviation: TE, thrombotic events.

Patients with a TE history were significantly more likely to experience a TE during the observation period (11/96, 11.5%) than patients with no history of TE (33/697, 4.7%; *p *= 0.014) (Table 2), which was supported by the cumulative incidence of TEs during the study (*p *= 0.003) (Figure 1). In contrast, age, sex, a history of BMF (i.e., aplastic anaemia [AA] and/or myelodysplastic syndromes [MDS]), and baseline LDH levels did not correlate with the occurrence of TEs during eculizumab treatment (Table 2).

**TABLE 2:** Characteristics of patients with or without TE occurrence during eculizumab treatment.

	Patients, *n* (%)			
Items	Total (*n = *794)^a^	Without TE (*n = *750)	With TE (*n = *44)	*p*‐value^b^
Age				
< 65 years	457	438 (95.8)	19 (4.2)	0.058
≥ 65 years	334	309 (92.5)	25 (7.5)	
Sex				
Male	403	380 (94.3)	23 (5.7)	0.878
Female	391	370 (94.6)	21 (5.4)	
AA and/or MDS (BMF)				
No	365	346 (94.8)	19 (5.2)	0.757
Yes	426	401 (94.1)	25 (5.9)	
History of TE				
No	697	664 (95.3)	33 (4.7)	0.014
Yes	96	85 (88.5)	11 (11.5)	
Baseline LDH, U/L				
< 1.5 × ULN	31	29 (93.5)	2 (6.5)	0.689
≥ 1.5 × ULN	740	699 (94.5)	41 (5.5)	

Abbreviations: AA, aplastic anaemia; BMF, bone marrow failure; LDH, lactate dehydrogenase; MDS, myelodysplastic syndrome; TE, thrombotic events; ULN, upper limit of normal.

^a^ Some items may not sum to the number in the total because of missing data.

^b^ Fisher's exact test.

Of the 44 patients who experienced a TE, six (13.6%) experienced multiple TEs under eculizumab treatment. A history of TE before eculizumab treatment was significantly associated with the cumulative incidence of multiple TEs during treatment (*p *= 0.003) (Table 3). In these patients, age, sex, AA and/or MDS (BMF) and baseline LDH levels did not correlate with the multiple TE occurrences during eculizumab treatment.

**TABLE 3:** Characteristics of patients who experienced multiple TEs during eculizumab treatment.

	Patients, *n* (%)^a^			
Items	Total (*n = *794)	No multiple TEs (*n = *788)	Multiple TEs (*n = *6)	*p*‐value^b^
Age				
< 65 years	457	453 (99.1)	4 (0.9)	1.000
≥ 65 years	334	332 (99.4)	2 (0.6)	
Sex				
Male	403	400 (99.3)	3 (0.7)	1.000
Female	391	388 (99.2)	3 (0.8)	
AA and/or MDS (BMF)				
No	365	362 (99.2)	3 (0.8)	1.000
Yes	426	423 (99.3)	3 (0.7)	
History of TE				
No	697	695 (99.7)	2 (0.3)	0.003
Yes	96	92 (95.8)	4 (4.2)	
Baseline LDH, U/L				
< 1.5 × ULN	31	30 (96.8)	1 (3.2)	0.219
≥ 1.5 × ULN	740	735 (99.3)	5 (0.7)	

Abbreviations: AA, aplastic anaemia; BMF, bone marrow failure; LDH, lactate dehydrogenase; MDS, myelodysplastic syndrome; TE, thrombotic events; ULN, upper limit of normal.

^a^ The denominator is the total number of each item.

^b^ Fisher's exact test.

### 3.4 TE Occurrence and Antithrombotic Agent Use

Of the 794 patients in this study, 207 (26.1%) used concomitant antithrombotic agents during eculizumab treatment (Table S1). More patients with a history of TE used concomitant antithrombotic agents (74.0%, 71/96) than those without a history of TE (19.5%, 136/697).

Of the 44 patients who experienced TEs during eculizumab treatment, 32 (72.7%) were on concomitant antithrombotic agents (Table 4). There were 63 prescriptions in total, 53 (84.1%) for anticoagulants and 10 (15.9%) for antiplatelet agents.

**TABLE 4:** Timing of antithrombotic agent administration in patients who presented with TEs during eculizumab treatment.

Items		Timing of antithrombotic agent use	Patients with TEs during eculizumab treatment, *n* (%)
History of TE	No *n = *22	Before TE occurrence	14 (63.6)
		After TE occurrence	6 (27.2)
		Unknown	2 (9.1)
	Yes *n = *10	Before TE occurrence	6 (60.0)
		After TE occurrence	3 (30.0)
		Unknown	1 (10.0)

Abbreviation: TE, thrombotic events.

Among patients without a history of TE, 14/22 (63.6%) were using antithrombotic agents before TE occurrence, while 6/10 (60.0%) of those with a history of TE were using antithrombotic agents before TE occurrence (Table 4).

**FIGURE 2:**
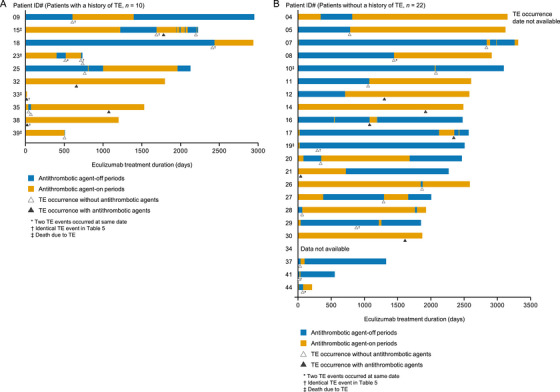
Timing of concomitant anticoagulation administration and TE occurrence during eculizumab treatment among patients (A) with and (B) without a history of TE. Abbreviation: TE, thrombotic events.

Regarding the timing of TE onset and the duration of antithrombotic agents during eculizumab treatment, TEs were more often observed during the antithrombotic agent off‐period (71.8%, 28/39) than during the on‐period (28.2%, 11/39) (Figure 2). Of the 32 patients with concomitant antithrombotic agent use, six (18.6%) died of TEs, of whom 4/10 (40%) had a history of TE and 2/22 (9.1%) did not.

### 3.5 Relationship between TE Occurrence and LDH

Of the 32 patients experiencing TEs during eculizumab treatment and receiving antithrombotic agents, 12 (37.5%, 13 events) had LDH data in the week before TE onset available (Table 5). LDH levels of ≥ 1.5 × ULN were observed in 8/13 TEs (61.5%). LDH levels 1 month before the onset of TE were the highest in patients with shunt thrombosis (1065 U/L), venous thrombosis in limb (967 U/L), disseminated intravascular coagulation (775 U/L), portal (689 U/L) and deep vein thrombosis (575 U/L). In three events (patients #3 pneumococcal pneumonia, #19 infection and #42 cellulitis), infectious AEs occurred before or at the same time as the onset of TE. For two events (patients #18 and #23), eculizumab had been given with a prolonged dosing interval. Table S2 shows the relationship between TE occurrence and eculizumab dosing. Of the 44 patients who experienced TEs, 11 (25.0%) had underlying conditions that increased their risk, such as a potentially triggering infection or a prolonged interval between doses of eculizumab. In addition, many patients experienced TEs just before the next dose of eculizumab.

**TABLE 5:** Relationship between TE occurrence and LDH levels (*n* = 12).

			Second to last LDH value before TE occurrence		Most recent LDH value before TE occurrence		Days from TE event to last eculizumab dosing^b^	
Patient ID #	History of TE	TE event	LDH, U/L	Days from TE event^a^	LDH, U/L	Days from TE event^a^		Note
03	No	Disseminated intravascular coagulation	183	−13	207	0	−12	Pneumococcal pneumonia as an AE was reported at TE onset
09	Yes	Cerebral infarction^c^	221	−33	257	0	−5	
15	Yes	Venous thrombosis in limb	790^d^	−70	967^d^	−7	−10	
18	Yes	Venous thrombosis in limb^c^	258	−42	241	0	−28	
19	No	Disseminated intravascular coagulation^c^	316	−56	775^d^	−7	−7	Infection as an AE was reported 8 days before TE occurrence
23	Yes	Cerebral infarction^c^	374^d^	−60	405^d^	0	−16	
29	No	Pulmonary embolism^c^	542^d^	−55	493^d^	−5	−13	
33	Yes	Haemorrhagic cerebral infarction^c^	288	−4	350^d^	0	−3	
38	Yes	Portal vein thrombosis^c^	1124^d^	−14	689^d^	−7	−7	
41	No	Deep vein thrombosis^c^	1935^d^	−9	575^d^	−1	−1	
42	Yes	Shunt thrombosis	N/A	N/A	1065^d^	−6	−6	
		Peripheral arterial occlusive disease	257	−14	283	0	−14	Cellulitis as AE at the onset of TE
43	No	Acute myocardial infarction	1793^d^	−13	297	0	−5	

Abbreviations: AE, adverse event; LDH, lactate dehydrogenase; N/A, not applicable; TE, thrombotic events; ULN, upper limit of normal.

^a^ Days = day of LDH measurement − day of TE event.

^b^ Days = day of last eculizumab dosing before TE event − day of TE event.

^c^ Identical TE event in Figure 2.

^d^ LDH ≥ 1.5 × ULN.

## 4 Discussion

In this sub‐analysis of an eculizumab PMS in Japan, the incidence of TE during eculizumab treatment was 1.50/100 patient‐years (54 events in 44/794 [5.5%] patients). This suggests that eculizumab treatment contributed to the prevention of TE in Japanese patients with PNH, although they were not abolished. The TEs often occurred alongside infections, prolonged dosing intervals and elevated LDH levels, potentially indicating incomplete terminal complement inhibition as one of their causes.

TE risk during PNH can be as high as 44% in untreated patients [4, 5]. However, a lower rate than in Western countries, of approximately 15%, has been reported in Japan [21]. One study reported annual TE incidences of 0.03 and 0.01/year (i.e., 3 and 1/100 patient‐years, respectively) for patients with untreated PNH with and without evidence of haemolysis, respectively [22]. Eculizumab has been shown to reduce the incidence of major adverse vascular events over 6 months from 3.9/100 patient‐years to 0.7/100 patient‐years [23].

It is well established that elevated LDH is a pivotal marker of IVH. If LDH is significantly increased above the ULN, there is persistent ongoing haemolysis and an elevated TE risk [24]. The Korean National PNH Registry reported that LDH ≥ 1.5 × ULN is associated with an increased risk of TEs and possibly mortality [4]. In the current analysis, a reasonable proportion (61.5%) of TEs occurred in patients with LDH ≥ 1.5 × ULN the week before the onset of the TE. High LDH values (∼1000 U/L) were observed in patients with shunt thrombosis and venous thrombosis in the limbs, suggesting that high LDH contributed to the development of TEs in these cases. Furthermore, 6/12 patients (50.0%) had LDH ≥ 1.5 × ULN at the second‐to‐last observation before TE occurrence. Therefore, LDH levels may be a useful biomarker for assessing TE risk among patients with PNH. Regular LDH monitoring and appropriate treatment, including eculizumab dose or dosing interval adjustment, may help prevent TE onset.

We previously reported that longer eculizumab dosing intervals (> 2 weeks) contributed to acute haemolysis regardless of infections [19]. In this sub‐analysis, 15.4% of TE events occurred with dosing intervals > 2 weeks. Pharmacokinetic‐related breakthrough haemolysis is known to occur due to insufficient serum levels in the effective range of C5 inhibitor treatment [25]. Compliance with dosing was one of the treatment burdens with eculizumab due to the 2‐week dosing interval. In these patients, LDH levels at TE onset were not dramatically elevated, but this may suggest that breakthrough haemolysis due to a prolonged dosing interval may contribute to the development of TEs. The current standard of care for PNH is ravulizumab, a long‐acting C5 inhibitor engineered from eculizumab with an extended dosing interval of 8 weeks [26]. In a recent 6‐year follow‐up of ravulizumab, major adverse cardiovascular event rates, including TEs, during treatment were well controlled and low throughout the study, both in complement inhibitor‐naïve patients and those previously treated with eculizumab (prior to study enrolment, 3.4–3.7/100 patient‐years; last follow‐up, 0.7–1.4/100 patient‐years) [27]. Furthermore, TEs observed in this study were not associated with breakthrough haemolysis events during ravulizumab treatment; these also resolved without modification of ravulizumab, with no reports of treatment discontinuation [27]. This suggests that sustained inhibition of IVH with long dosing intervals reduces the rate of breakthrough haemolysis and may prevent further thrombosis risk and mortality compared with eculizumab [9].

In this analysis, a history of TE was significantly correlated with TE occurrence during eculizumab treatment, consistent with a previous report [28]. In the general population, ageing, smoking, diabetes and hypertension are risk factors for TEs [29], and they may also be independent risk factors for TE in this PNH population. Of those risk factors, only age data were available for analysis in this study, and while there was no significant relationship between age and the occurrence of TEs during eculizumab treatment, patients with a history of TE at baseline were significantly older than those without a history of TE. In general, TE associated with these risk factors is widely recognized as being caused by the involvement of atherosclerosis [30]; however, the mechanism of TEs in PNH is quite different. While it is not yet fully understood, free haemoglobin caused by IVH can trigger TE, directly inducing platelet aggregation or via nitric oxide adsorption [5]. Moreover, several reports showed that TEs in PNH predominantly occur in the venous vessels [13, 31]. In the current analysis, about 30% of patients with TE had arterial thrombosis, and 76.5% of patients were > 65 years of age (37.5% of patients with venous thrombosis) (data not shown). This suggests that arterial thrombosis might develop independently of the pathogenesis of PNH. Gurnari et al. [30] reported that patients with Type 2‐dominant PNH clone size were at higher risk of developing TEs. We did not analyse the Type 2 PNH clone size ratio; however, monitoring this may be important in identifying high‐risk groups for PNH‐related TEs.

The risk–benefit profile of concomitant antithrombotic agent use in patients receiving anti‐complement therapy has not been fully evaluated [32]. In this PMS, 26.1% of patients received concomitant antithrombotic agents, most before the onset of TE, and TEs occurred more often in patients in the antithrombotic agent off‐period than those in the on‐period (71.8% vs. 28.2%). In an International PNH Registry study, approximately 30% of patients received antithrombotic therapy at baseline [33], which is similar to the current findings. However, in our study, the percentage of concomitant antithrombotic agent use tended to be much higher in patients with a history of TE. Contrary to our findings, some reports suggest that TEs in PNH cannot be prevented by the concomitant use of antithrombotic therapy [5, 34], which indicates the complexity of managing TEs in this condition. Furthermore, we observed cases of recurrent thrombosis after discontinuation of the antithrombotic agent.

Our results suggest that there is some benefit to concomitant use of antithrombotic agents in managing TE risk regardless of a history of TE. The risk of thrombosis associated with PNH has been dramatically reduced with the advent of C5 inhibitor treatments [27, 35, 36], while thrombosis caused by other factors may have decreased with the use of antithrombotic agents in these patients. Because the present analysis does not differentiate between PNH‐related thrombosis and thrombosis caused by other factors, the results should be interpreted with caution. However, while C5 inhibitors have substantially improved the prognosis of PNH patients compared with that of untreated PNH patients [27], a clinical gap remains. Thus, there may be some rationale for the use of concomitant antithrombotic agents in the comprehensive management of patient prognosis. Another important point is that concomitant antithrombotic agents do not completely prevent thrombosis, and further evidence is needed to advance our understanding of this.

This study's limitations include the absence of a control group, missing data and inadequate or incomplete follow‐up because of the observational design. Furthermore, given the nature of the data collection, there is a potential for incomplete or selective reporting of events. We did not assess the potential fatal outcomes of TEs. As information regarding TEs was based on AE data, it is impossible to determine whether all the TEs were PNH‐derived. Thus, the incidence of these events relating to PNH may be overestimated. In addition, no information was collected in this PMS on common risk factors for TEs, such as smoking, diabetes and hypertension, nor did the data specify the purpose of using antithrombotic agents. Due to the PMS's observational nature, caution is warranted when extrapolating results to broader populations, as the patient cohort may not represent all individuals with PNH.

Overall, the current findings contribute to the growing body of evidence supporting the benefit of terminal C5 inhibition in reducing thrombotic risk in Japanese patients with PNH. Eculizumab appears to help prevent TEs in PNH, but it cannot eliminate their occurrence entirely. TEs appear to be associated with higher LDH levels, infections and long dosing intervals, potentially indicating incomplete terminal complement inhibition at the time of TE. Concurrent eculizumab use to prevent TEs in PNH is likely clinically useful, especially in patients with a history of TE, but further evaluation is required. Comprehensive studies with control groups and standardized data collection on risk factors are needed to improve generalizability and inform clinical decision‐making for managing TEs in PNH.

## Author Contributions

H.H. conceptualized the study, analysed the data and drafted the manuscript. A.S. designed the study. None of the authors was directly involved in data collection, which was performed by Alexion Pharmaceuticals. All authors participated in data interpretation, and critical review and approval of the final manuscript.

## Funding

This study was funded by Alexion Pharma G.K., Tokyo, Japan.

## Ethics Statement

The study was conducted in compliance with Good Post‐marketing Surveillance Practice guidelines, which the Pharmaceuticals and Medical Devices Agency provides to comply with submitted PMS data integrity standards for regulatory submission documentation. All patients who were included in this PMS provided consent for publication.

## Conflicts of Interest

Takayuki Ikezoe has served as a consultant for Alexion, Chugai, Asahikasei Pharma and Novartis; received speaker honoraria from Alexion, Chugai, Asahikasei Pharma and Novartis; received support for attending meetings and/or travel from Alexion, Chugai, Asahikasei Pharma and Novartis; and is a Director of the Japanese Society of Hematology, and the Japanese Society of Thrombosis and Hemostasis. Tatsuya Kawaguchi has received speaker honoraria from Sobi and Novartis. Hideo Hayashi and Akihiko Shimono are employees of Alexion Pharma GK and hold stock or stock options in Alexion, AstraZeneca Rare Disease. Jun‐ichi Nishimura has served as a consultant for Alexion, Chugai, Roche, Sobi, Asahikasei Pharma and Novartis; and received speaker honoraria from Alexion, Chugai, Roche, Sobi, Asahikasei Pharma and Novartis.

## Supporting information




**Supplementary Table 1**: Characteristics of patients at baseline stratified by history of TE.
**Supplementary Table 2**: Relationship between TE occurrence and eculizumab dosing.
**Supplementary Figure 1**: Patient disposition.

## Data Availability

Alexion, AstraZeneca Rare Disease will consider requests for disclosure of clinical study participant‐level data, provided that participant privacy is assured through methods like data de‐identification, pseudonymization, or anonymization (as required by applicable law) and if such disclosure was included in the relevant study informed consent form or similar documentation. Qualified academic investigators may request participant‐level clinical data and supporting documents (statistical analysis plan and protocol) pertaining to Alexion‐sponsored studies. Further details regarding data availability and instructions for requesting information are available in the Alexion Clinical Trials Disclosure and Transparency Policy at https://www.alexionclinicaltrialtransparency.com/data‐requests/
