# Supplementary material for: Thrombotic Events in Patients With Paroxysmal Nocturnal Haemoglobinuria Using Eculizumab: A Post‐Marketing Surveillance Sub‐Analysis
Source: EJHaem. 2026 May 26;7(3):e70287. doi: 10.1002/jha2.70287 (PMC13240519; doi:10.1002/jha2.70287)
Supplement: Supplementary file 1 — Supplementary Table 1: Characteristics of patients at baseline stratified by history of TE. Supplementary Table 2: Relationship between TE occurrence and eculizumab dosing. Supplementary Figure 1: Patient disposition. [file JHA2-7-e70287-s001.pdf]

Supplementary Materials

Thrombotic Events in Patients with Paroxysmal Nocturnal Haemoglobinuria Using Eculizumab: a Post-Marketing Surveillance Sub-Analysis

Takayuki Ikezoe[1], Tatsuya Kawaguchi[2], Hideo Hayashi[3], Akihiko Shimono[3], Jun-ichi Nishimura[4]

[1]Department of Hematology, Fukushima Medical University, Fukushima, Japan

[2]Department of Medical Technology, Kumamoto Health Science University, Kumamoto, Japan

[3]Medical Affairs Division, Alexion Pharma G.K., Tokyo, Japan

[4]Department of Hematology and Oncology, Osaka University Graduate School of Medicine, Osaka, Japan

Supplementary Table 1 Characteristics of patients at baseline stratified by history of TE.

Items	Safety analysis set *N* = 794	History of TE: Yes *n* = 96	History of TE: No *n* = 697	*p*-value[a]
Age, years				
Mean (SD)	55.4 (18.9)	61.8 (16.0)	54.5 (19.1)	<0.001
Median (range)	59.0 (9.0–90.0)	67.5 (22.0–86.0)	57.0 (9.0–90.0)	
Female, *n* (%)	391 (49.2)	47 (49.0)	344 (49.4)	1.000
History of AA and/or MDS (BMF), *n* (%)				
Yes	426 (53.7)	54 (56.3)	372 (53.4)	0.663
No	365 (46.0)	42 (43.8)	322 (46.2)	
Unknown	3 (0.4)	0	3 (0.4)	
History of thromboembolism, *n* (%)				
Yes	96 (12.1)	96 (100.0)	-	-
With concomitant antithrombotic agent	71 (8.9)	71 (74.0)	-	-
Without concomitant antithrombotic agent	25 (3.1)	25 (26.0)	-	-
No	697 (87.8)	-	697 (100.0)	-
With concomitant antithrombotic agent	136 (17.1)	-	136 (19.5)	-
Without concomitant antithrombotic agent	561 (70.7)	-	561 (80.5)	-
Unknown	1 (0.1)	0	0	
Treatment duration, median days (range)	1389.0 (1.0–4215.0)	1523.5 (8.0–3543.0)	1351.5 (1.0–4215.0)	0.397
Incidence of TE, /100 Patient-years	1.50	4.08	1.11	

[a]Between patients with history of TE and those without history of TE

Abbreviations: AA, aplastic anaemia; BMF, bone marrow failure; MDS, myelodysplastic syndrome; SD, standard deviation; TE, thrombotic events.

Supplementary Table 2 Relationship between TE occurrence and eculizumab dosing.

Patient ID#	History of TE	TE event	Eculizumab last dosing (days from TE)	Haemolysis and infection−related adverse event (days from TE)
01	No	Shunt thrombosis	−13	
02	No	Cerebral infarction	−8	
03	No	Disseminated intravascular coagulation	−12	Pneumococcal pneumonia (0)
04	No	Haemorrhoids thrombosed	Data not available	
05	No	Deep vein thrombosis	−6	
06	No	Acute myocardial infarction	−2	
07	No	Deep vein thrombosis	−12	
08	No	Pulmonary embolism	−4	
		Venous thrombosis limb	−4	
09	Yes	Cerebral infarction	−5	
10	No	Cerebral infarction	−1	Bronchitis (−24), Upper respiratory infection (−22), Sepsis (−15), Skin infection (−13), Sepsis shock (−4)
11	No	Thrombosis	−3	
12	No	Deep vein thrombosis	−11	
13	No	Placental infarction	Data not available	
14	No	Venous thrombosis limb	−4	
15	Yes	Venous thrombosis limb	−10	
		Budd−Chiari syndrome	−14	
		Myocardial infarction	−17	
16	No	Renal vein thrombosis	−2	
17	No	Heparin−induced thrombocytopenia	−14	
18	Yes	Venous thrombosis limb	−28	
19	No	Disseminated intravascular coagulation	−7	Infection (−8)
20	No	Deep vein thrombosis	−14	
21	No	Thrombosis	−8	Haemolysis (0), Enteritis infections (0)
22	No	Disseminated intravascular coagulation	Data not available	

23	Yes	Portal vein thrombosis	−12	
		Hepatic vein thrombosis	−12	
		Cerebral infarction	−16	
		Cerebral infarction	−27	
24	No	Cerebral infarction	−2	
25	Yes	Deep vein thrombosis	−14	Haemolysis (0)
26	No	Peripheral arterial occlusive disease	−22	
27	No	Lacunar infarction	−6	
28	No	Deep vein thrombosis	−7	
29	No	Pulmonary embolism	−13	
30	No	Venous thrombosis limb	−10	
31	Yes	Disseminated intravascular coagulation	Data not available	
32	Yes	Disseminated intravascular coagulation	−3	Haemolysis (−30), Sepsis (0)
33	Yes	Haemorrhagic cerebral infarction	−3	
34	No	Disseminated intravascular coagulation	Data not available	
35	Yes	Cardiac ventricular thrombosis	−12	
		Cardiac ventricular thrombosis	−14	
		Thrombosis	−8	
36	No	Cerebral infarction	−9	
37	No	Cerebral infarction	−4	
38	Yes	Portal vein thrombosis	−7	
39	Yes	Cerebrovascular accident	−13	
40	No	Acute myocardial infarction	−10	
41	No	Deep vein thrombosis	−1	
42	Yes	Shunt occlusion	−6	
		Peripheral arterial occlusive disease	−14	Cellulitis (0)
43	No	Acute myocardial infarction	−5	
44	No	Pulmonary embolism	−5	
		Deep vein thrombosis	−5	

Abbreviation: TE, thrombotic events.

Supplementary Figure 1 Patient disposition.

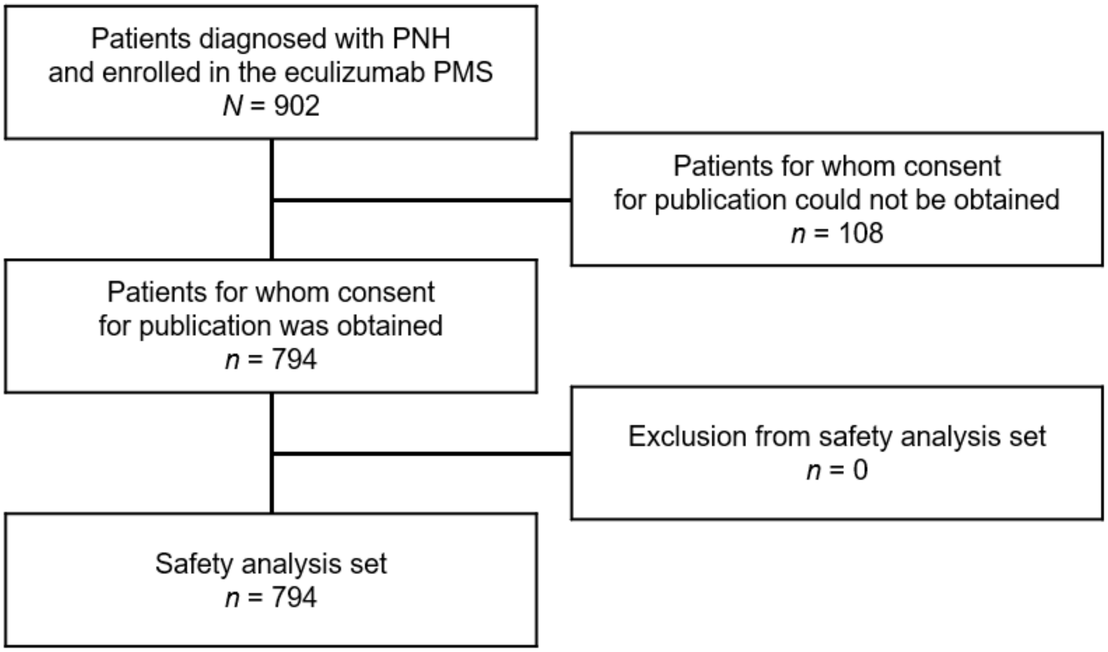

Abbreviations: PNH, paroxysmal nocturnal haematuria; PMS, post-marketing surveillance.
